# A Robust and Affordable Table Indexing Approach for Multi-isocenter Dosimetrically Matched Fields

**DOI:** 10.7759/cureus.1270

**Published:** 2017-05-23

**Authors:** Amy Yu, Benjamin Fahimian, Lynn Million, Annie Hsu

**Affiliations:** 1 Department of Radiation Oncology, Stanford University School of Medicine

**Keywords:** table indexing, tli, safety, dosimetric match fields, shift error

## Abstract

Purpose

Radiotherapy treatment planning of extended volume typically necessitates the utilization of multiple field isocenters and abutting dosimetrically matched fields in order to enable coverage beyond the field size limits. A common example includes total lymphoid irradiation (TLI) treatments, which are conventionally planned using dosimetric matching of the mantle, para-aortic/spleen, and pelvic fields. Due to the large irradiated volume and system limitations, such as field size and couch extension, a combination of couch shifts and sliding of patients are necessary to be correctly executed for accurate delivery of the plan. However, shifting of patients presents a substantial safety issue and has been shown to be prone to errors ranging from minor deviations to geometrical misses warranting a medical event. To address this complex setup and mitigate the safety issues relating to delivery, a practical technique for couch indexing of TLI treatments has been developed and evaluated through a retrospective analysis of couch position.

Methods

The indexing technique is based on the modification of the commonly available slide board to enable indexing of the patient position. Modifications include notching to enable coupling with indexing bars, and the addition of a headrest used to fixate the head of the patient relative to the slide board. For the clinical setup, a Varian Exact Couch^TM^ (Varian Medical Systems, Inc, Palo Alto, CA) was utilized. Two groups of patients were treated: 20 patients with table indexing and 10 patients without. The standard deviations (SDs) of the couch positions in longitudinal, lateral, and vertical directions through the entire treatment cycle for each patient were calculated and differences in both groups were analyzed with Student's t-test.

Results

The longitudinal direction showed the largest improvement. In the non-indexed group, the positioning SD ranged from 2.0 to 7.9 cm. With the indexing device, the positioning SD was reduced to a range of 0.4 to 1.3 cm (p < 0.05 with 95% confidence level). The lateral positioning was slightly improved (p < 0.05 with 95% confidence level), while no improvement was observed in the vertical direction.

Conclusions

The conventional matched field TLI treatment is error-prone to geometrical setup error. The feasibility of full indexing TLI treatments was validated and shown to result in a significant reduction of positioning and shifting errors.

## Introduction

**Figure 1:**
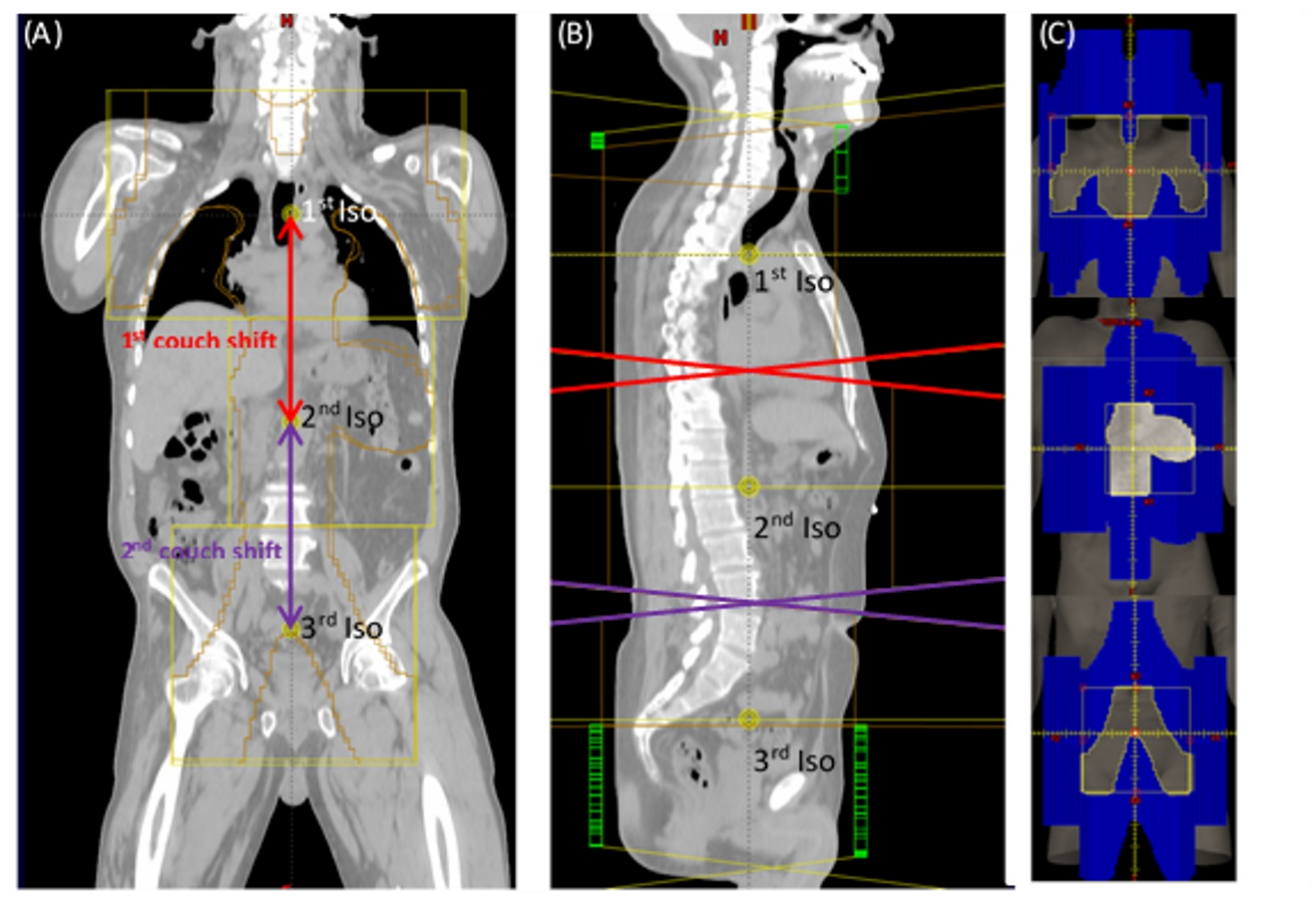
The field matching for total lymphoid radiation (TLI) treatment. (A) Anterior-posterior view of the field with the multileaf collimator (MLC) outlined in orange. In order to achieve the dosimetric match, the absolute couch shift is required. The first couch shift is the distance between the first isocenter and second isocenter. The second couch shift is the distance between the second isocenter and third isocenter. (B) Lateral view of three TLI fields with the diverging border (red and purple). (C) MLC arrangement for each field.

The ability to obtain a reproducible patient setup and accurate positioning within the treatment coordinate system is of critical importance for the safe and accurate delivery of radiotherapy. This aspect is emphasized in dosimetrically matched fields requiring exact shifts of abutting fields with different isocenters [1-3]. With indexed immobilization devices, tolerance tables can be established in order to minimize the shift errors [4-5] and to reduce patient setup errors. Among dosimetrically matched treatment planning techniques, total lymphoid irradiation (TLI) presents unique challenges. Due to restrictions in the couch extension and patient height, patient sliding may be necessary, in addition to couch shifts, making robust couch indexing particularly challenging. Conventional planning of fields for TLI treatment consists of three distinct isocenter sites – mantle, para-aortic/spleen, and pelvis fields [6-7] (Figure 1).

The non-indexed delivery of treatments incorporating multiple isocenters and dosimetrically matched fields is associated with a number procedural difficulties and safety issues. First, without indexing, it is not discernable how the anatomical sites were set up and treated based on non-indexed couch positions recorded in the record and verify system. Second, couch shifts solely based on values calculated by therapists prior to beam-on allow for the introduction of human error and are a common cause geometrical misses. Third, for tall patients (if the shift from the first isocenter to the third isocenter is more than 51 cm), the couch can reach its longitudinal shift limit, leaving it to the therapy team to slide the patient superiorly to align the pelvis field to the correct isocenter location; since the patient anatomy may be stretched in this scenario, even if the shift is correct, the patient’s geometry is no longer the same so the dosimetric match is no longer achievable. Fourth, without indexing the treatment, the physicists are not able to assign a couch position tolerance limit to detect deviations in the patient setup and to prevent the possibility of mistreatments [5]. It is a challenge to index TLI treatments because of the limited length of the treatment couch and its travel range. In order to enhance treatment safety, the aim of this study is to develop and evaluate a robust and affordable approach to dosimetrically index matched field treatments and to avoid the geometrical misses and shift errors. 

## Materials and methods

### Immobilization and indexing device 

An illustration of the TLI field arrangement is presented in Figure 1. 

**Figure 2:**
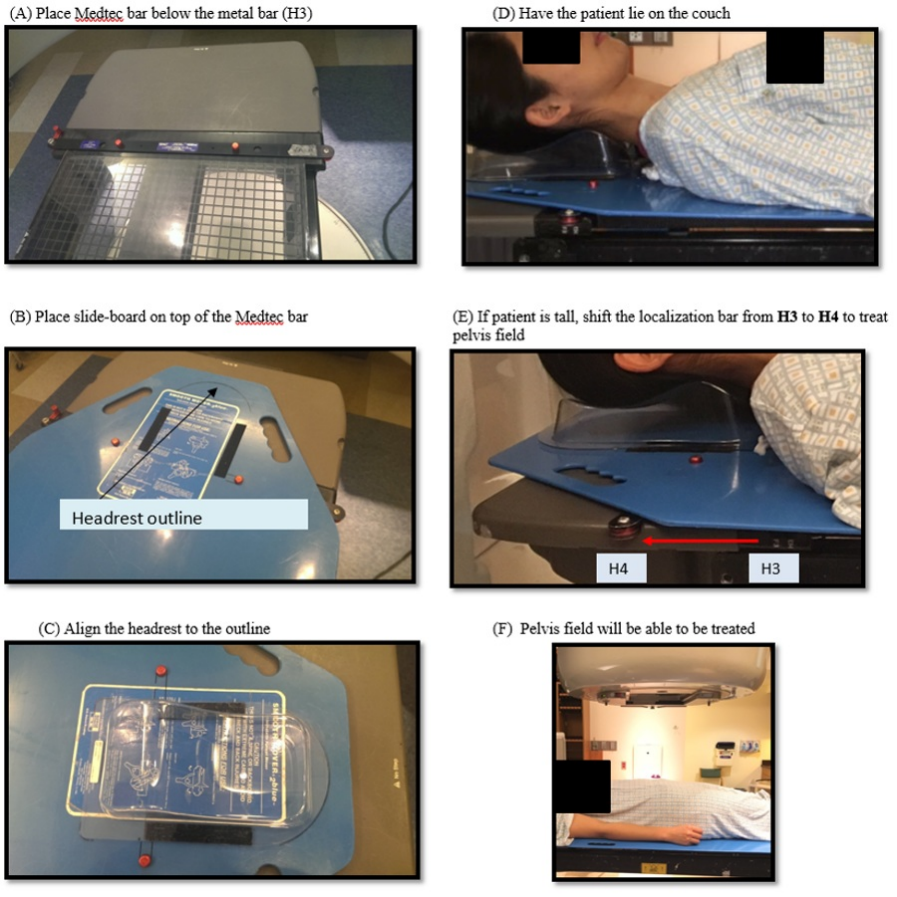
Steps for assembly of the indexing device and subsequent treatment of tall patients

The utilized immobilization device was constructed based on a modification of a conventional slide board, Smooth-Mover^TM^ (Radiation Products Design, Inc., Albertville, MN). It is made of polyethylene construction with six slotted hand grips. Two holes were drilled in the superior area for the patient’s headrest. The position of the headrest was selected so the indexing bar is not interfering with the first (mantle) field. The distance and the size of the two holes were matched to the two pins of the Exact Lok-Bar (CIVCO Medical Solutions, Coralville, IA), which is used to index the slide board to the couch top. For treatment, the Exact Lok-Bar was put to the predetermined notch (H3) to avoid the first (mantle) field going through the metal bar. The Timo Headrest (CIVCO Medical Solutions, Coralville, IA) was attached to the slide board with Velcro so the therapist can use a different size of Timo Headrest based on the patient anatomy. The position of the Timo Headrest was outlined beforehand (Figure 2B). 

For the third (pelvis) field, if the patient’s height requires an unattainable longitudinal shift (beyond the table travel limit of 160 cm), therapists can unlock the Exact Lok-Bar and shift the slide board to the superior notch (H4) (Figure 2E). Since the distance between each notch on the couch is fixed (14 cm), the couch shift between spleen field and pelvis field can be calculated by Eq. 1:

$Couch\; Shift (cm)=Distance\; between\; two\; isocenters (cm)-14 cm$                                                    (Eq. 1)  

### Evaluation of dosimetric effect of the device

The dosimetric effect of the indexed device is evaluated based on the recommendations of the American Association of Physicists in Medicine Task Group 176 [8]. Attenuation of the slide board was measured with Farmer ion chamber (PTW, Freiburg, Germany) for 15 MV (n = 3). An ion chamber was placed at a depth of 5 cm in solid water (100 cm source axis distance (SAD), 10 x 10 cm field size). The attenuation of the slide board (A) was calculated by Eq. 2:

$A=1-\frac{x}{y}$                                                                                                                                                            (Eq. 2) 

where x is the *electrometer reading taken with the slide board* and y is the *electrometer reading taken without the slide board. *

The skin dose was evaluated with the Optically Stimulated Luminescence Detectors (OSLD) (Landauer, Inc., Glenwood, IL) for 15 MV (n = 3). The experimental setup was 100 cm source skin distance (SSD) and 10 x 10 cm field size with OSLDs placed on the couch or on the slide board with 5 cm backscatter solid water to mimic the treatment setup. A computed tomography (CT) scan of solid water with the slide board was acquired and imported into treatment planning system (TPS) with the analytical anisotropic algorithm to estimate the attenuation and the skin dose with the plan parameters corresponding to the measurement conditions. 

### Clinical workflow

Two slide boards were constructed: one for simulation and the other for treatment. The patient was simulated at CT with the modified slide board and the Exact bar was placed at H3. The patient’s head was positioned on the Timo Headrest. The midline of the patient was aligned with the sagittal laser to ensure the patient was straight. Prior to the treatment, the therapist took verification images at every isocenter (mantle, spleen, and pelvis) to ensure the accuracy of the patient setup, with the couch positions acquired and recorded in the system. Tattoos were placed on the patient: one set of tattoos were placed at the second isocenter (spleen), and two midline tattoos were placed at the first and the third isocenters in order to straighten the patient, as well as to serve as reference positions for daily treatments. For tall patients, when the required shift for the pelvis isocenter reached the couch extension limit, the board was slid superiorly from H3 to H4 (a fixed distance of 14 cm) and the remaining couch shift was calculated by Eq. 1. A summary of steps for the procedure is shown in Figure 2.

### Patient selection and data analysis

Twenty patients were treated with this immobilization device and 10 patients were treated with the conventional setup. For tall patients, defined as patients for which the shift from the first isocenter to the third isocenter is greater than the couch extension limit, patient sliding is necessitated; within the indexed group, two patients were tall and needed to slide from H3 to H4 to treat the pelvic field (Figure 2E), and within the non-indexed group, three patients were tall and needed to slide superiorly to treat the pelvic field. For both groups, the fractionation regimen was 1.2 Gy per fraction with a total of 10 treatments [9]. Patients were randomly selected from our database and the non-indexed patients were all treated before the indexed patients were treated (before the indexing device was implemented). The SDs of couch position and p-value through entire treatment (10 fractions) were calculated for each direction with the Student's t-test, i.e., longitudinal, lateral, and vertical directions, to determine the significance of improvement of this immobilization technique. In order to systematically develop the clinical tolerance limit for indexed treatments, a value 1.96 SD of the couch positions, representing 95% of the area of a normal distribution, was used to establish the daily tolerance limits for indexed TLI treatment.

## Results

**Figure 3:**
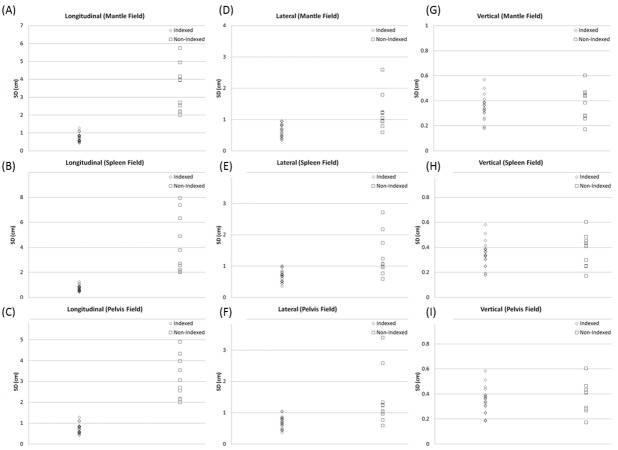
The standard deviation of the couch positions across the entire treatment without and with the implementation of the indexing technique The standard deviation (SD, cm) without (squares, n = 10) and with (diamonds, n = 20) the implementation of the indexing technique for mantle, spleen, and pelvic fields. The SD in longitudinal (A-C) and lateral (D-F) directions reduces significantly. The SD in the vertical (G-I) direction remains similar. P-values of Student’s t-test are 10^-22^, 10^-9^, and 0.16 for longitudinal, lateral, and vertical directions, respectively.

The SDs of couch positions across the entire treatment are calculated (Figure 3) for both non-indexed (n = 10) and indexed (n = 20) groups in longitudinal, lateral, and vertical directions (Student's t-test, p-values are 10^-22^, 10^-9^, and 0.16, respectively). With the conventional setup (non-indexed), the SD of 10 fractions ranged from 2.0 to 7.9 cm and 0.6 to 3.4 cm in longitudinal and lateral directions, respectively (Figure 3A-3F). Using the new immobilization technique, the SD ranged from 0.4 to 1.3 cm and 0.4 to 1.0 cm in longitudinal and lateral directions, respectively (Figure 3A-3F). The variations in the vertical direction are similar for both non-indexed and indexed treatments and ranged from 0.2 cm to 0.6 cm (shown in Figure 3G-3I). The average total length of the three fields and the shift between the first isocenter and the third isocenter for the tall patients was 74.8 cm (± 2.8 cm) and 52.8 cm (± 4.6 cm), respectively. The average total length of the three fields and the shift between the first isocenter and the third isocenter for the regular patients was 66.2 cm (± 3.4 cm) and 42.3 cm (± 2.8 cm), respectively. 

**Figure 4:**
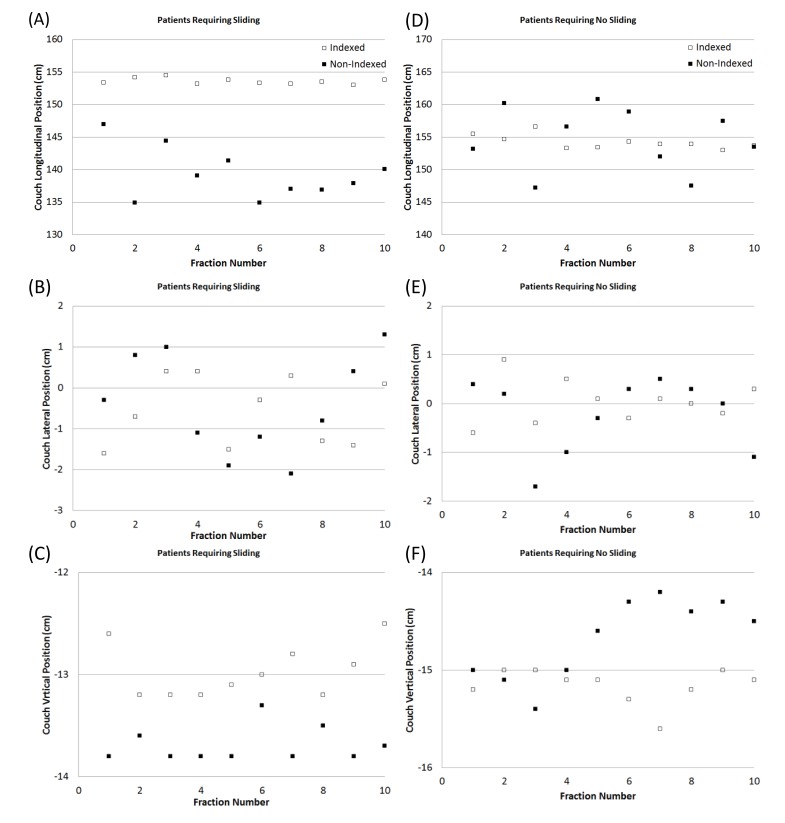
Individual cases (patients) representing the worst cases for indexed and non-indexed treatments (A-C) Patients are too tall to treat the pelvic field. In order to treat the pelvic field, the patient will need to be moved superiorly. (D-F) For patients of regular height, no sliding is required. Indexed couch positions are presented with white squares and non-indexed couch positions are presented with black squares.

The couch positions of the entire 10 fractions (the worst case was presented) for tall and regular height patients are shown in Figure 4 for indexed and non-indexed treatments. As expected, the variation of couch positions for 10 fractions with indexed treatment remains the same for both situations (tall and regular height patients) through the treatment as compared the non-indexed treatment for the pelvis field in the longitudinal directions. The variation of the couch positions in lateral and vertical are comparable between the tall patient and the patient with regular height. The attenuation of the slide board was 1.1 % measured by the ion chamber and 0.9 % calculated by TPS. The skin dose increased from 40% to 76% measured by OSLD due to the bolus effect from the slide board. If the prescribed dose is 12 Gy, the skin dose increased from 4.8 $\pm$ 0.003 Gy to 9.1 $\pm$ 0.004 Gy. The skin dose with the slide boards was also confirmed with TPS (skin dose increased from 4.09 Gy to 8.1 Gy). 

## Discussion

The observed SD range of the longitudinal table position (2 - 7.9 cm) is representative of the variation of the daily non-indexed couch position through the entire treatment, not the target variation. The large variation of the couch position for patients in the non-indexed group was because (1) therapists laid the patient on a different location on the couch every day because there was no indexed device to guide them otherwise, and (2) for patients requiring an additional sliding in order to treat the pelvic field, larger variations were observed because the shift would be therapist-dependent, differing in the amount from day to day. With properly indexed devices, therapists can predictably set the patient up, such that a tolerance limit can be developed, thus minimizing shifting errors. Couch indexing minimizes errors in radiotherapy, especially for dosimetrically matched treatment fields, which can result in overdose or underdose around the field junction region due to incorrect shift or improper setup. 

**Figure 5:**
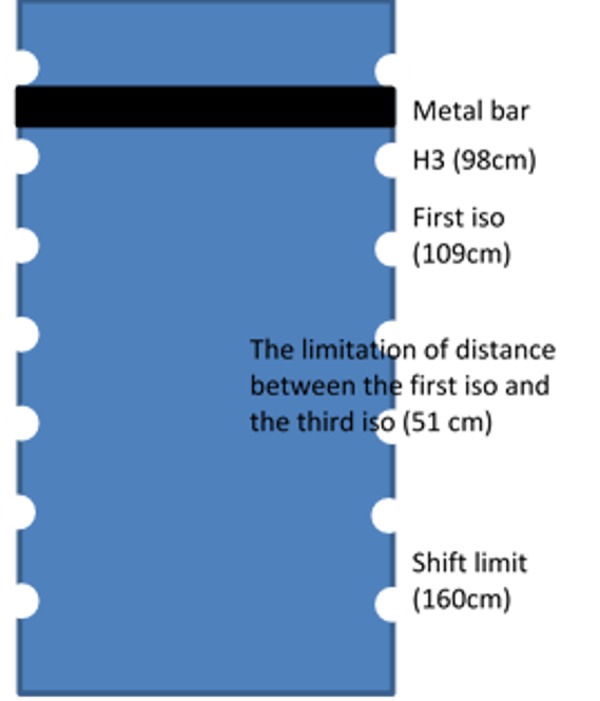
The limitation of distance between the first isocenter and the third isocenter The longitudinal couch shift limitation is dependent on the field setup and the patient’s anatomy. If the shift from the first isocenter to the third isocenter is more than 51 cm, it is very possible to reach the couch longitudinal extension limit.

If the shift from the first isocenter to the third isocenter is more than 51 cm, it is possible to reach the longitudinal shift limit, depending on the field setup and the patient’s anatomy. The longitudinal couch position for H3 is 98 cm and the limitation for longitudinal is 160 cm. The first treatment field cannot go through the metal bar so the superior border of the mantle field has to be below H3. If a conservative assumption is made about the length of the mantle field (22 cm), this would result in the mantle isocenter being located relative to the couch at ~109 cm (98 cm + 11 cm). Due to the couch extension limitation of 160 cm, the distance between mantle isocenter and the pelvis isocenter needs to be less than 51 cm (Figure 5). For tall patients, this may not be achievable. Therefore, during sliding the patient without the indexed device, it is easy to stretch and deform their posture. In this scenario, the dosimetric match is no longer valid. Using the proposed technique, the patient lies on the rigid slide board with the relative posture of the patient similar between before and after the patient is shifted. Based on the variations of couch positions in this study, couch position tolerance limits for the TLI treatments were set to ± 2 cm in longitudinal and lateral directions and ± 1 cm in the vertical direction for indexed patients.

Since the slide board is available in most clinics, with the proposed solution, the slide board not only can be used to transfer patients but also can be used to index TLI treatment without extra cost and storage space. Since the holes are very small compared to the entire board, the rigidity of the board is not expected to be changed. Indexed dosimetrically matched treatments can thus be made safer for the patient due to the predictability of the couch position before the treatment and availability of tolerance table. Moreover, physicists can identify mistreatments and perform dose reconstruction during the treatment cycle via a weekly chart check using the record and verify system [10-11]. The couch has tolerance limits which can be assigned to each treatment field to prevent the possibility of mistreatment and improve the setup accuracy [5]. This technique not only can be used for the TLI treatment but also for other multiple isocenter field matched treatments (e.g. craniospinal treatments). There are commercial products on the market to serve similar indexing purpose, including Delta Couch (Varian Medical Systems, Inc, Palo Alto, CA), Vac-LokTM cushions (MEDTEC, Orange City, IA) [12-13], and couch inserts for enhanced patient positioning. However, these products are either expensive or require storage space, and most importantly, the limitation of couch shift still remains unresolved for treating tall patients (the treatment requires an additional shift to treat the pelvic field). The Varian Exact IGRT couch does not have the metal bar and allows using the table from an earlier longitudinal direction, although it requires large capital expenses to upgrade the couch. The attenuation of the slide board is less than the carbon fiber couch top [14]. Skin dose increases from 40% to 76% for a posterior 15 MV beam. However, a typical TLI treatment contains only posterior 15 MV beams, i.e., no oblique beam to result in higher skin dose, and it was also demonstrated in this study that it is able to be modeled with TPS. 

## Conclusions

Extended volume treatments require the dosimetric matching of multiple fields with different isocenters, which are prone to shifting errors and positioning errors. An indexed device has been developed and demonstrated to significantly reduce the variation of the couch positions in the longitudinal and lateral directions as compared to the non-indexed treatment. This method is easily adaptable and affordable as it comprises the modification of readily available equipment in most clinics. With this approach, physicists can easily evaluate the setup variation based on the couch position recorded in the system via weekly chart QA during the course. Therapists can calculate the couch position as the second verification before beam on. Furthermore, the safety and the reproducibility of the TLI treatment can be significantly improved with a tight couch position tolerance limit, which also works for tall patients. This study shows the proof of the concept that, with the slide board indexing, the indexed TLI treatment is feasible without large capital expenses.
